# The Formation of Glycerol Oligomers with Two New Types of End Groups in the Presence of a Homogeneous Alkaline Catalyst

**DOI:** 10.3390/polym11010144

**Published:** 2019-01-16

**Authors:** Dawid Kansy, Kornelia Bosowska, Krystyna Czaja, Anna Poliwoda

**Affiliations:** Faculty of Chemistry, University of Opole, Oleska 48, 45-052 Opole, Poland; kornelia.bosowska@uni.opole.pl (K.B.); Krystyna.czaja@uni.opole.pl (K.C.); Anna.poliwoda@uni.opole.pl (A.P.)

**Keywords:** glycerol, oligoglycerol, oligomerization, catalysis, mechanism, polyglycerols

## Abstract

The purpose of this work was to synthesize and characterize oligoglycerols with the chains of more than four repeating units. Those oligoglycerols may have some interesting applications, among others, as polyoxyalkylation starters. The glycerol oligomerization process was carried out during 12 h, at 230 °C, under the pressure of 0.4 bar, with the use of sodium carbonate as a homogeneous basic catalyst; various concentrations of the catalyst in the reaction medium were used. The reaction products were analyzed with the use of direct infusion electrospray ionization mass spectrometry (ESI-MS), nuclear magnetic resonance (^13^C NMR) and Fourier transform infrared spectroscopy (FTIR) techniques. Based on the analytical findings, the compositions of the obtained product mixtures and the structures of oligoglycerols present in individual fractions were determined. The effect of catalyst concentration on the composition of the post-reaction mixture was observed. Moreover, in addition to the conventional linear oligomers (α,α-oligoglycerols), two new types of the oligomers were for the first time detected in the post-reaction mixture: one with two hydroxyl groups and the other with a carboxylate group at the α-carbon atom.

## 1. Introduction

The presently growing interest in fuels from renewable sources is driven by the efforts of government authorities, which results from the depletion of oil resources. Biofuels can be produced inter alia by transesterification of fats with methyl alcohol or ethyl alcohol. About 10 wt % of the feed to that process will be converted to a by-product; i.e., to glycerol [1]. The demand for glycerol (propane-1,2,3-triol) is growing at an average rate of about 7% per year and its production is estimated to reach 6 million tons per year in 2025 [2].

**Figure 1:**
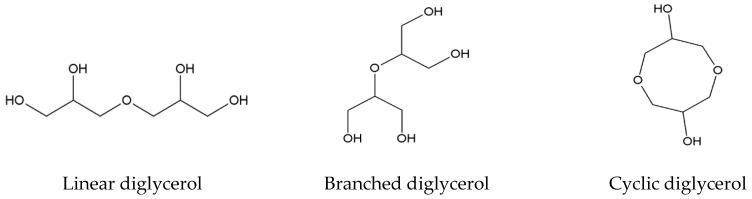
Examples of diglycerol structures, which can be formed in the glycerol etherification process.

Glycerol can undergo numerous chemical reactions, i.e., oxidation, dehydration, reduction, selective transesterification, etherification, etc. Within the etherification reaction, two glycerol molecules undergo condensation to yield the most simple oligomer, i.e., diglycerol. The diglycerol product may be linear, branched or cyclic, dependent on the hydroxyl groups (at the primary or secondary carbon atoms) which are involved in the glycerol condensation reaction (Figure 1). Further stages of the condensation reaction may also produce tri-, tetra- and higher oligomers. Oligoglycerols with 2–3 repeating units have many applications in the cosmetic and pharmaceutical industries, while oligomers with four and more repeating units can be used as components for the production of fatty acid esters or as starters for the polyoxyalkylation process. It is a great challenge for scientists to control the glycerol oligomerization process to selectively produce the desirable oligomer or the desirable fraction of oligoglycerols, which can be done principally by selecting the catalyst and its properties carefully. Oligomerization of glycerol can be carried out with the use of homogeneous or heterogeneous, acidic or basic catalysts. Heterogeneous catalysts are easy to recover from the reaction mixture, hence they can be recycled and used many times. However, as compared to homogeneous catalysts, they have lower activity and, in some cases, lower thermal stability, requiring longer reaction times and high reaction temperatures. Mainly zeolites were used as acidic heterogeneous catalysts. The use of the acidic homogeneous catalyst, such as sulfuric acid, made the oligomerization process faster, but its selectivity was inferior. Hydroxides, carbonates and alkali metal oxides were tested as basic catalysts (both homogeneous and heterogeneous). Although hydroxides are stronger bases than carbonates, potassium carbonate turned out to be a better catalyst than potassium hydroxide because of its better solubility in glycerol and in the reaction product. In general, the glycerol oligomerization processes in the presence of basic catalysts were slower, yet more selective than in the presence of acid catalysts [3,4,5,6,7,8,9,10].

This report presents research on the glycerol oligomerization process with the use of sodium carbonate, which is readily soluble in glycerol and which makes a homogeneous basic catalyst; various concentrations of the catalyst in the reaction medium were tested. The syntheses of oligoglycerols, which have been described in the professional literature, usually produce the products which are composed of four constitutional glycerol units (mers) at most. The purpose of this work is to produce glycerol oligomers with the average molecular weight over 350 g/mol, i.e., those containing more than four glycerol units, and to determine their structures.

## 2. Materials and Methods

### 2.1. Materials

Oligomerizations were carried out using glycerol, anhydrous (≥99.5%), p.a. M = 92.10 g/mol purchased from the company Chempur (Piekary Śląskie, Poland); sodium carbonate, anhydrous (≥99.5%), p.a. M = 105.99 g/mol from POCH (Gliwice, Poland) and nitrogen (Messer, Chorzów, Poland). For purification, the following products were used: ethyl acetate (≥99.5%), p.a. M = 88.11 g/mol, propan-2-ol (≥99.5%), p.a. M = 60.10 g/mol from Protolab (Słupsk, Poland), sodium hydroxide (≥99.8%), p.a. M = 39.99 g/mol, sulfuric acid (96%), p.a. M = 98.08 g/mol from POCH (Gliwice, Poland) and ethoxyethane (≥99.8%), p.a. M = 74.12 g/mol from Chempur (Piekary Śląskie, Poland).

### 2.2. Synthesis of Oligoglycerols

The oligoglycerols were produced through the oligomerization reaction, with the use of basic homogeneous catalysis. The reactions were conducted in a 500 mL three-necked flask on which a thermometer, an accessory nitrogen bubbling unit and a condenser for reduced-pressure distillation were installed. The glycerol oligomerization reactions were catalyzed by Na_2_CO_3,_ which was used at various concentrations. Initially, 400 mL of glycerol and a suitable amount of the catalyst were placed in the flask. The reaction mixture was heated up to 55 °C with nitrogen bubbling through it for 14 min. Then, the flow of nitrogen was stopped since the nitrogen stream was responsible for distillation of the substrate at higher temperatures. The condensation process was conducted under agitation with the magnetic bar stirrer for 12 h at 230 °C and under the pressure of 400 mbar. After that time, the post-reaction mixture was cooled down to room temperature and the obtained product was weighed.

### 2.3. Purification and Isolation of Products

**Figure 2:**
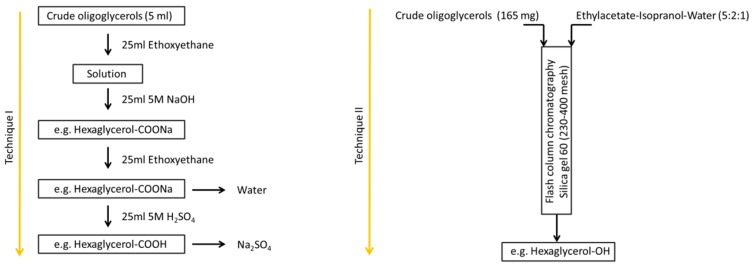
An exemplary scheme for the purification of oligoglycerols.

Purification of the crude oligoglycerols was carried out using two techniques (Figure 2). The first purification method was the use of a reactive extraction. 5 mL of oligoglycerol were dissolved in 25 mL of ethoxyethane and extracted with 25 mL of 5 M NaOH (1:1) (*v*/*v*). The extracting mixture was then cooled from room temperature to 10 °C to avoid the emerging microemulsion. After cooling, the alkaline phase was separated from the organic phase and extracted again with ethoxyethane, where the organic phase was in turn extracted with 5 M H_2_SO_4_ in the ratio (1:1) (*v*/*v*). The result was an acid fraction of the oligomerization product that has been subjected to ^13^C NMR analysis.

Method II consisted of the use of flash column chromatography filled with silica gel (230–400 mesh, Merck Kieselgel 60). A mixture of ethyl acetate-isopranol-water (5:2:1) (*v*/*v*) was used as the mobile phase system. The conditions were carried out on normal phase silica gel (TLC silica gel 60 F 254 Merck, Darmstadt, Germany) as the stationary phase with ethyl acetate-isopranol-water (5:2:1) (*v*/*v*) as the mobile phase. Retention factors (R_f_) of glycerol, diglycerol, triglycerol and tetraglycerol fractions obtained from our chemical reactions were 0.55, 0.45, 0.33 and 0.26, respectively. After completion of the separation, the eluent was removed in vacuo. The separated products of column chromatography were characterized by ^13^C NMR.

### 2.4. Analysis of Oligoglycerols

The oligomerization products were analyzed with the use of the following techniques: mass spectrometry (MS) micrOTOF-QII instrument with ESI, from Bruker Daltonik GmbH; nuclear magnetic resonance (^13^C NMR) Ultrashield 400 MHz instrument from Bruker, Rheinstetten, Germany; and Fourier-transform infrared spectroscopy (FT-IR)–Nicolet 6700 instrument from Thermo Scientific, San Jose, CA, USA. Also, viscosity of the product was measured with the use of the Alpha series rotational viscometer from Fungilab, equipped with the TL5 spindle. The ESI-MS analyses were performed with the microOTOF-Q II system (Bruker Daltonics, Bremen, Germany) fitted with an electrospray source (ESI). The mass spectra were recorded in the range of 50–1500 *m*/*z* in the negative ionization ion mode. The analyzed samples were diluted with the water:methanol (1:3 *v*/*v*) mixtures and then directly infused into the ESI source at the flow rate of 3 µL min^−1^ using a micro syringe pump. High purity nitrogen was used both as the drying and nebulizing gas. The following ESI source conditions were applied: capillary temperature 180 °C, sheath gas flow rate 4 L min^−1^, spray voltage −2800 V.

## 3. Results and Discussion

The effect of the concentration of the basic catalyst (sodium carbonate) on the conversion degree in the glycerol oligomerization reaction and on the quality and composition of the products was studied. The reactions gave liquid products with their colouration changing from yellow to brown for the increasing catalyst concentration. The conversion for the tested catalyst concentrations in the range of 0.67–3.33 wt % in each case was above 90%. In turn, darker and darker colouration, and first of all higher and higher viscosity of the post-reaction mixture was indicative for the growing degree of polymerization at the higher concentrations of the catalyst (Table 1). This relationship is consistent with the known theoretical dependence of the degree of polymerization on the polycondensation conversion.

**Figure 3:**
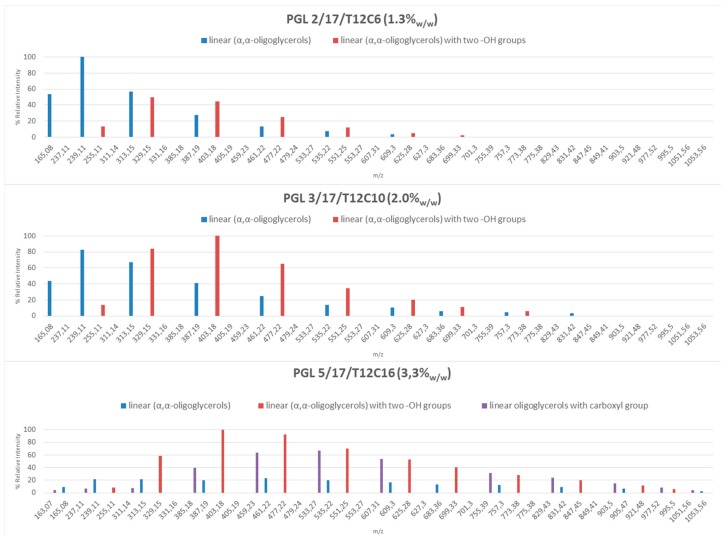
The effect of catalyst concentration on the observed mass distribution for the synthesized oligoglycerols, as determined from the ESI-MS spectra of the analyzed samples (*m*/*z* = [M − H]^−^).

The reaction products were then analyzed by ESI(-)MS mass spectrometry to investigate their molecular weights and structures. Electrospray ionization mass spectrometry already become an alternative approach in monitoring the products of glycerol oligomerization, especially to detect a number of specific properties [11,12]. The ESI-MS spectra of the obtained products were analyzed to show that the compositions of individual mixtures were dependent on the concentration of the catalyst used. In each case, the analyzed post reaction samples turned out to be the mixtures of oligoglycerols with various chain lengths. Generally, the increase of the sodium carbonate concentration affected the chain growth of the obtained oligoglycerols (Figure 3).

**Figure 4:**
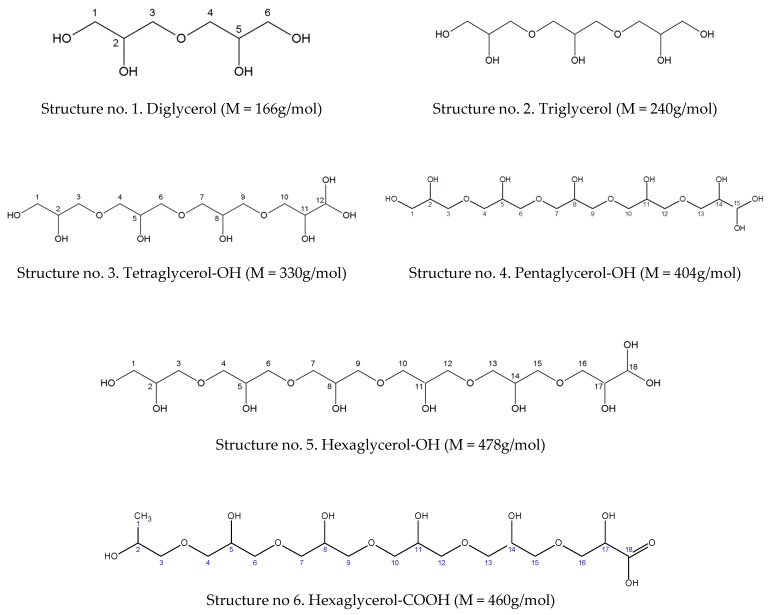
Examples of typical and non-typical structures of oligoglycerols.

Three types of linear oligomers were found in the reaction products. There were conventional linear oligomers (α,α-oligoglycerols), and additionally the presence of new oligomers with atypical structures of oligoglycerol macromolecules with two hydroxyl groups or a carboxyl group at the α carbon atom was found. It is interesting that no presence of cyclic forms was observed in the obtained mixtures. For example, when Nguyen R. et al. used K_2_CO_3_ [12], significant amounts of smaller oligomers with cyclized repeating units were detected. In the case of our linear α,α-oligoglycerols, the oligomers with 2–6 repeating units were mostly determined. Furthermore, this concerned the products obtained with the use of low catalytic concentrations (≤2.0 wt %). The identified oligomers [M − H]^−^: *m*/*z* 165.08; 239.11; 313.15; 387.19) correspond to typical structures of di-, tri-, tetra- and penta-glycerol exhibited a higher relative intensity (over 50%). Interestingly, any further increase of the Na_2_CO_3_ concentration in the reaction mixtures resulted in a decrease of the amounts of the classical linear α,α-oligoglycerols, in favour of the increasing amounts of linear oligoglycerols but with two –OH groups at the α carbon atom. At the catalyst concentrations of over 3.3 wt %, the significant presence of such oligomers with 4–9 repeating units was confirmed. Moreover, oligomers with the carboxyl group at the α carbon atom were also visible. The molecular weights of the obtained atypical oligomers with two –OH or with the carboxyl group at the α-carbon atom were determined in the range from 256 Da to 1070 Da. However, the products which consisted of 5, 6 or 7 glycerol units were the dominant forms. The examples of conventional and atypical structures of oligoglycerols are presented below (Figure 4).

**Figure 5:**
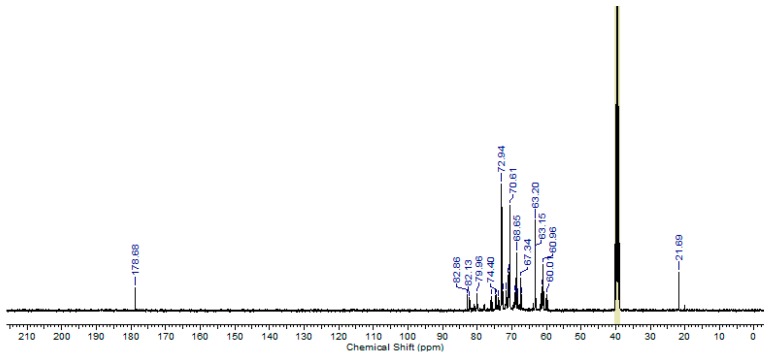
^13^C NMR spectrum of PGL 5/17/T12C16 post-reaction mixture. (100 MHz, DMSO-*d*_6_).

The structures of the obtained oligoglycerols, with the new group at the α-carbon atom, have also been confirmed by ^13^C NMR analysis. In the first stage, post-reaction mixtures were analyzed. An example of the spectrum for PGL 5 is shown in Figure 5.

The signals appearing in the spectrum at the chemical shifts of 60–63 and 67–75 ppm can be assigned to carbon atoms in the following groups: –CH_2_–OH, >CH–OH, –CH_2_–O– and >CH–O–. There are also two signals at the chemical shifts of 82 and 179 ppm which are specific for the α-carbon atom substituted by two hydroxyl groups and for carbon of the carboxyl group.

**Figure 6:**
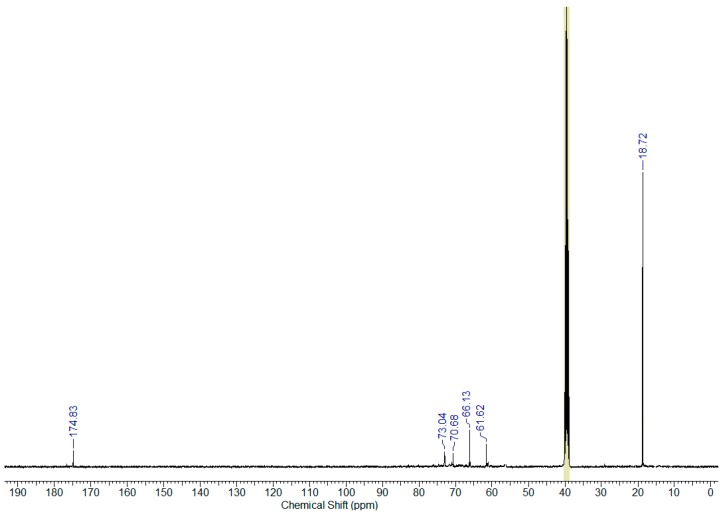
^13^C NMR spectrum of PGL 5/FK—fraction of PGL5/17/T12C16 with –COOH. (100 MHz, DMSO-*d*_6_).

Then, the acid fraction derived from the PGL 5/17/T12C16 obtained as a result of the reactive extraction process marked as PGL 5/FK was analyzed by ^13^C NMR (Figure 6).

**Figure 7:**
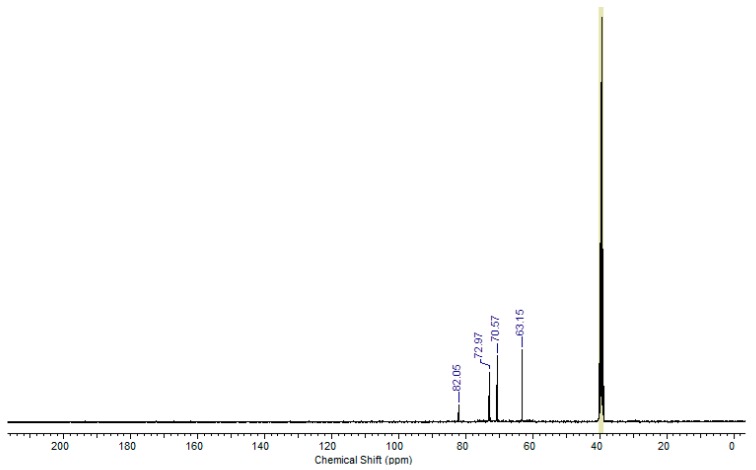
^13^C NMR spectrum of PGL 5/FC—fraction of PGL5/17/T12C16 with double –OH. (100 MHz, DMSO-*d*_6_).

**Figure 8:**
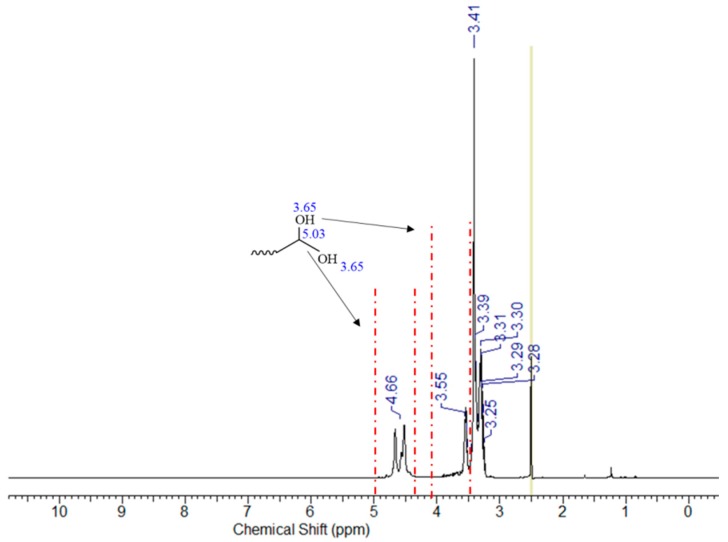
^1^H NMR spectrum of PGL 5/FC—fraction of PGL5/17/T12C16 with two –OH. (400 MHz, DMSO-*d*_6_).

In this ^13^C NMR spectrum, the signals appearing at the chemical shifts of 61.62; 66.13; 70.68 and 73.04 ppm can be assigned to carbon atoms in the following groups: –O–**C**H_2_–, CH_3_–**C**H(OH)–, –O–**C**H_2_–CH(OH)– and –CH_2_–**C**H(OH)–CH_2_–. There are also signals at the chemical shifts of 18.72 and 174.83 ppm, which are specific for the carbon in –CH_3_ and the carbon atom of the carboxyl group. On the other hand, the fraction obtained with the use of flash column chromatography was analyzed by ^13^C NMR, the results of which are shown in the spectrum (Figure 7) marked as PGL 5/FC sample. In this ^13^C NMR spectrum the signals appearing at the chemical shifts of 63.15; 72.97; 70.57 ppm can be assigned to carbon atoms in the following groups: –O–CH_2_–, CH_2_–CH(OH)–CH_2_, CH(OH)–CH_2_–O. There is also a signal at the chemical shift of 82.05, which is specific for the carbon atom with two groups of –OH. Moreover, for the fraction obtained by flash chromatography, the ^1^H NMR analysis was additionally made (Figure 8). The results of this analysis confirmed the presence of two hydroxyl groups at the α carbon, where the characteristic signals at 4,66 ppm and 3,55 ppm for the proton associated with alpha carbon and for protons of hydroxyl groups attached to alpha carbon are visible. 

The values of chemical shifts determined on the basis of the NMR spectra were also compared with the theoretically specified values and both are summarized in Table 2.

**Figure 9:**
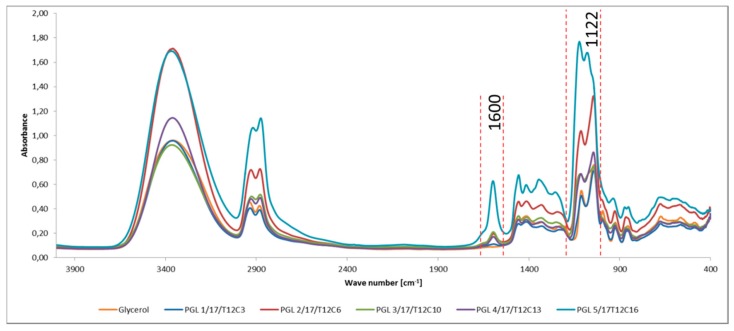
FTIR spectra (film between KBr) for the glycerol oligomerization products. The spectrum for the raw material (glycerol) was also presented for reference.

The presence of –COOH or two –OH groups at the α-carbon atom in the oligoglycerols obtained was also confirmed by the FTIR analysis (Figure 9). The band which is observed in the spectrum at the wave number of 1600 cm^−1^ is specific for the carboxylate ion [13,14]. Moreover, the band at 1111 cm^−1^ can be attributed to the presence of two –OH groups at the α-carbon atom. In turn, the bands in the FTIR spectrum typical of polyglycerols are: the band at the wave number of 1290–1100 cm^−1^ and 2962–2853 cm^−1^ corresponds to the stretching vibrations of C–O–C group and of CH and CH_2_, respectively. Finally, the stretching vibrations band for the –OH group intermolecularly associated through the hydrogen bonding is at the wave number of 3400–3200 cm^−1^.

**Figure 10:**
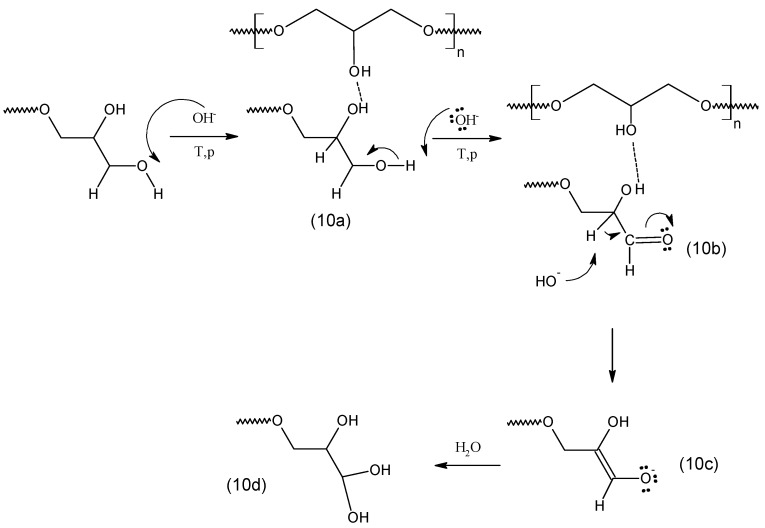
Proposed mechanism of formation of two hydroxyl groups.

**Figure 11:**
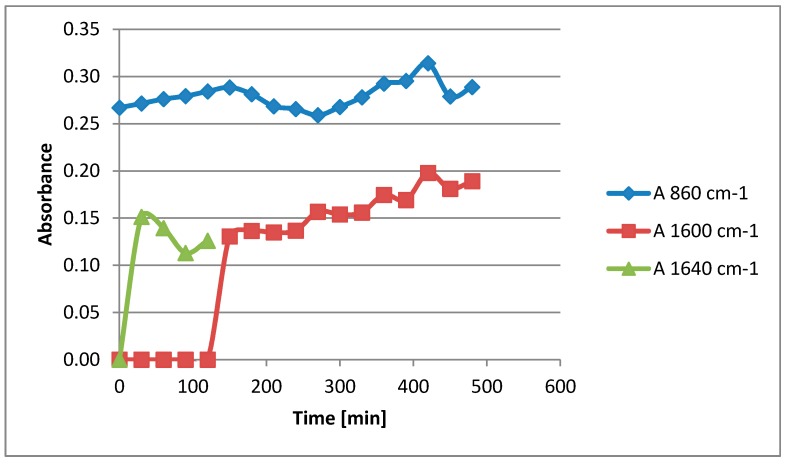
A plot of absorbance of the reaction time of the etherification of glycerol.

The oligoglycerols found in the reaction products with the –COOH group and the additional –OH group at the α-carbon atom arise as a result of the end of the chain-growth reaction according to various mechanisms. COOH-terminated oligoglycerols are likely formed in a strongly alkaline environment by the elimination of β-hydrogen from the oligoglycerol molecule, followed by the addition of CO_2_ from the catalyst to the β-carbon atom and the simultaneous separation of the aldehyde group (as in the Kolbe-Schmitt reaction). In turn, the formation of oligoglycerols with a geminal OH group proceeds according to the mechanism, the scheme of which is given in Figure 10. Accordingly, the end of the oligoglycerol chain growth occurs initially according to the nucleophilic Sn2 substitution mechanism (10a), by cleaving the proton from the hydroxyl group connected with the α carbon. It should be remembered that the reaction takes place at high temperature, under reduced pressure in a strongly alkaline environment, and the hydroxyl group connected to β-carbon is further stabilized by hydrogen bonding. As a result, further de-protonation in a strongly alkaline environment (10b) leads to the formation of a vinyl bond between α and β carbon atoms (10c) as previously described by Gilbert [15]. The proposed mechanism confirms the results of FTIR (Figure 11), which showed that together with the etherification reaction of glycerol, the absorbance of the C=CO bond at 1640 cm^−1^ is increased, and at a wavelength of 860 cm^−1^ derived from the C=C bond. The mentioned vinyl bond is also observed in the MS spectrum (Figure 3) at a molecular weight of e.g., 312 g/mol. Finally, a water molecule is attached to the double bond formed between α and β (10c) to give the geminal diol (10d).

## 4. Conclusions

This report presents the catalytic glycerol oligomerization process which yields oligoglycerols with the molecular weights higher than 350 g/mol, i.e., containing more than four glycerol units.

The glycerol feed which was used in the study contained water at the level below 0.5%. The oligomerization experiments demonstrated that the conversion was high (over 90%) in each case for the tested catalyst concentrations in the range of 0.67–3.33 wt %. The products with the growing molecular weights were obtained for the increasing concentrations of the homogeneous basic catalyst in the oligomerization reaction medium.

The analyses (with the use of ESI-MS, NMR and FTIR techniques) of the oligomerization products revealed the presence of typical oligoglycerols with various chain lengths and the presence of non-typical ones, which had not been described before, and which had one additional -OH group at the α-carbon atom or were terminated with the carboxyl group. The structures of these products were suggested and confirmed. Finally, the termination mechanisms for the chain-growth reaction were suggested for glycerol oligomerization, which lead to the formation of such atypical products.

## Figures and Tables

**Table 1:** Results of glycerol oligomerization reaction catalyzed by sodium carbonate; process conditions: 230 °C, 400 mbar and 12 h (PGL—Polyglycerol).

Concentration of the Catalyst C_cat_ [wt %]	Symbol	Viscosity at 80 °C [mPa·s]
0.67	PGL 1/17/T12C3	63.0
1.33	PGL 2/17/T12C6	221.1
2.0	PGL 3/17/T12C10	504.7
2.67	PGL 4/17/T12C13	286.4
3.33	PGL 5/17/T12C16	3757.2

**Table 2:** Chemical shift values for the signals determined from the ^13^C NMR analysis versus those calculated for hexaglycerol with two –OH groups and hexaglycerol with the carboxyl group at the α-carbon atom.

Oligomer	No. of Carbon Atom	^13^C NMR Theoretical [ppm]	^13^C NMR Measured [ppm]
(Structure no. 5) Hexaglycerol-OH NMR measured—Figure 7	1;4; 7; 10; 13; 16	61.50	63.15
	2; 5; 8; 11; 14;17	73.40	72.97
	3; 6; 9; 12; 15	71.50	70.57
	18	89.90	82.05
(Structure no. 6) Hexaglycerol-COOH NMR measured—Figure 6	1	18.20	18.72
	2	66.30	66.13
	3; 6; 9; 12; 15	71.50	70.68
	4; 7; 10; 13; 16	61.50	61.62
	5; 8; 11; 14; 17	73.40	73.04
	18	174.40	174.83
